# A Case of Thrombectomy for Loeffler’s Endocarditis

**DOI:** 10.7759/cureus.64384

**Published:** 2024-07-12

**Authors:** Takanobu Kimura, Akira Takeuchi, Takeru Nakamura, Koki Tamaoka, Hiroshi Tsuneyoshi

**Affiliations:** 1 Department of Cardiovascular Surgery, Shizuoka General Hospital, Shizuoka, JPN

**Keywords:** adult cardiac surgery, cardiac tumor, thrombectomy, eosinophilic endocarditis, loeffler’s endocarditis

## Abstract

Loeffler’s endocarditis, characterized by eosinophilic infiltration leading to thrombus formation and fibrosis in the ventricle, is associated with severe complications, such as embolism and heart failure. While anticoagulation and steroids are standard treatments, surgical thrombectomy is rarely reported. This is a case report of a 74-year-old man presented with an abnormal electrocardiogram. Echocardiography revealed a 38 × 29 mm mass in the left ventricular apex, and blood studies revealed hypereosinophilia, leading to a diagnosis of Loeffler’s endocarditis. Despite warfarin treatment, the thrombus persisted. The left ventricular intracardiac thrombus exhibited significant mobility, and we decided to perform a thrombectomy via a trans-left ventricular approach. After the surgery, steroid therapy was initiated. The patient recovered without recurrence of the thrombus or deterioration in cardiac function. Left ventricular thrombectomy for Loeffler’s endocarditis is considered a beneficial option to prevent thrombosis.

## Introduction

Loeffler’s endocarditis is a severe condition that can cause fatal embolism and heart failure [1,2]. Since it was defined by Loeffler et al. in 1936, many cases have been reported [1-4]. Inflammation of the endocardium, triggered by an increase in eosinophils, results in thrombus formation in the left ventricle and fibrosis of the left ventricular myocardium, consequently leading to impaired left ventricular wall motion [3]. The main causes of death in Loeffler’s endocarditis are embolism and heart failure, and the control of embolism is a critical factor in the treatment of Loeffler’s endocarditis [3]. The primary treatment involves anticoagulation therapy with warfarin and steroid administration, but the resolution of thrombosis usually takes a long time, and it is difficult to prevent thromboembolic events even with anticoagulation therapy [2,3]. However, there are few reports of cases in which surgical thrombectomy was performed. In this report, we present a case of Loeffler’s endocarditis with a highly mobile left ventricular thrombus, posing a high risk of embolism, where thrombectomy was performed to prevent embolism. Postoperatively, the eosinophil count, which initially decreased, increased again. Therefore, steroid therapy was initiated. The steroids were effective, resulting in a rapid decrease in eosinophil count, with no recurrence of intracardiac thrombus or progression of myocardial fibrosis observed. While steroids and anti-coagulation remain the fundamental treatment for Loeffler’s endocarditis, surgical thrombectomy is an important treatment option for preventing fatal embolism in this condition.

## Case presentation

**Figure 1:**
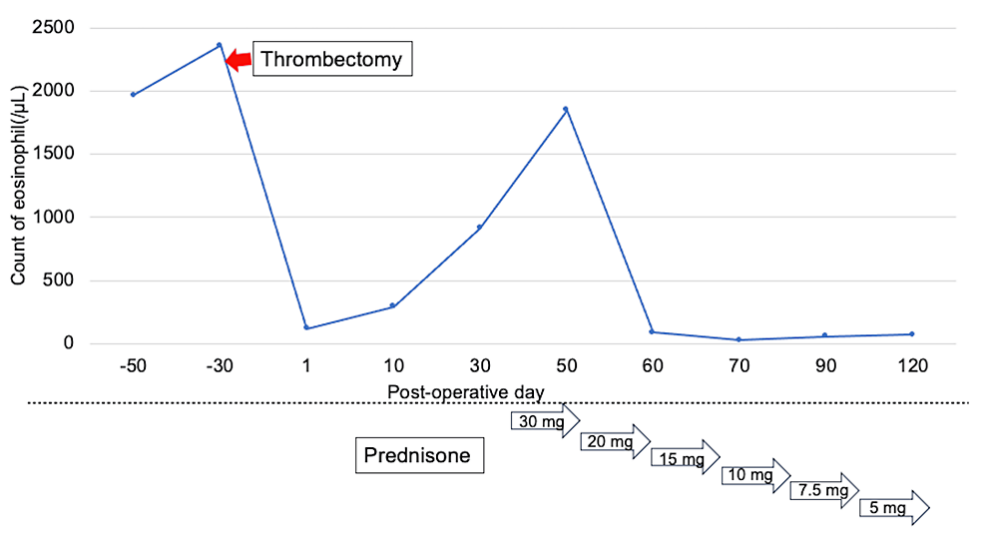
Eosinophil counts before and after thrombectomy; response to prednisone treatment Preoperatively, the eosinophil count was elevated. Following the thrombectomy, it temporarily decreased but increased again. After starting steroids, the eosinophil count quickly decreased and has remained low.

A 74-year-old man was noted to have an abnormal electrocardiogram during a health examination. Subsequent echocardiography revealed a mass in the apex of the left ventricle, and he was referred to our cardiology department. Two blood samples taken more than one month apart revealed elevated eosinophil counts of 1,968/μL and 2,363/μL, leading to the diagnosis of hypereosinophilia (Figure 1). No abnormalities were observed in the coagulation-fibrinolysis system, inflammatory markers, or tumor markers.

**Figure 2:**
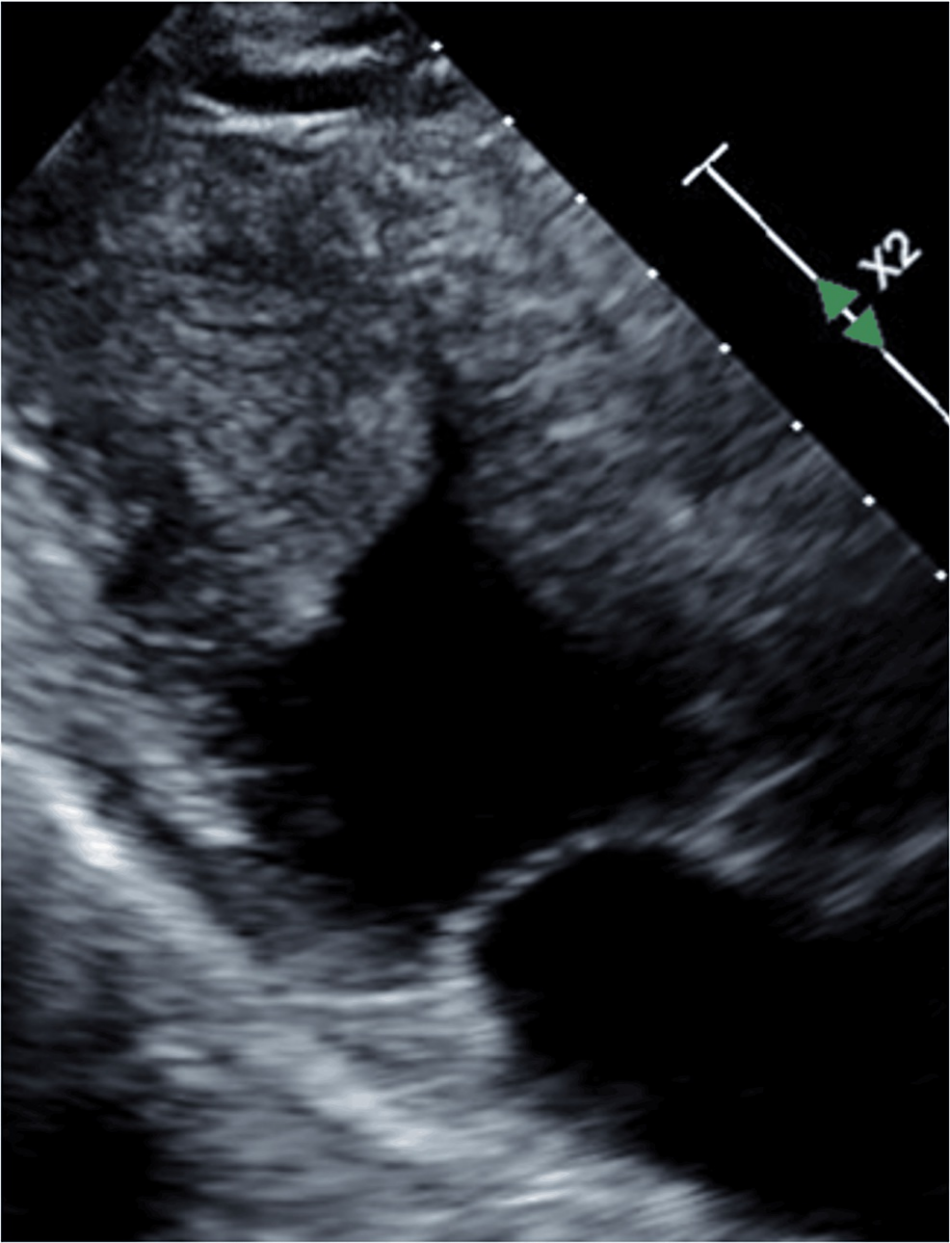
Echocardiographic findings Echocardiography revealed a mobile mass in the left ventricular apex.

The electrocardiogram showed negative T waves in II, III, aVF, and V4-V6. Echocardiography revealed a mobile 38 × 24 mm isobaric, echogenic, and aseptic mass in the apex lumen of the left ventricle (Figure 2). There was no Doppler effect inside the mass.

**Figure 3:**
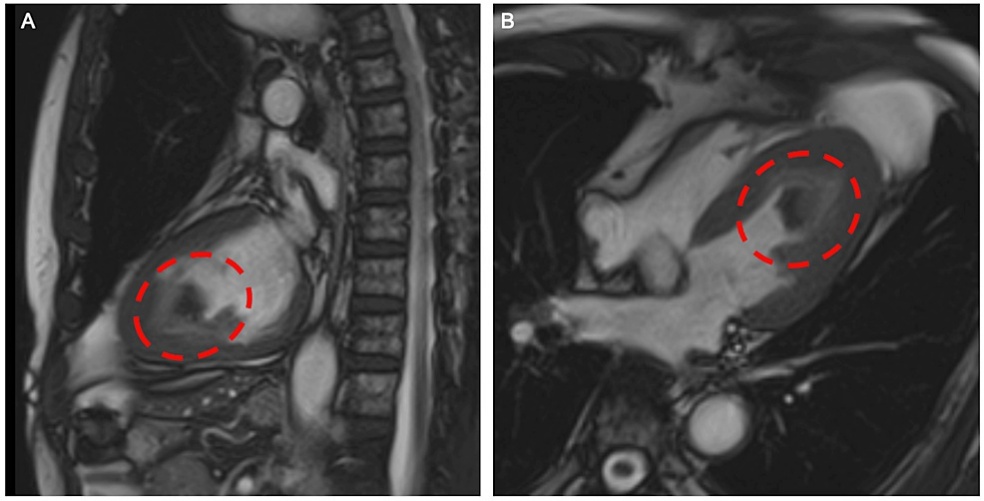
Cardiac MR image The red circle indicates the thrombosis in the sagittal (A) and horizontal (B) sections. A mobile mass with a low signal density on T1WI and T2WI was observed in the apex of the left ventricle; dynamic contrast showed no contrast effect.

The ejection fraction was 44% with apex hypokinesis. Contrast-enhanced CT showed a contrast-deficient area in the apex of the left ventricle, and there was no significant stenosis in the coronary arteries. There were no signs of embolism or tumor in other organs and no significant lymph node enlargement. A cardiac MRI showed a low-signal-intensity mass in the apex of the left ventricle on T1WI and T2WI (Figure 3). Cine mode revealed mobility of the mass.

**Figure 4:**
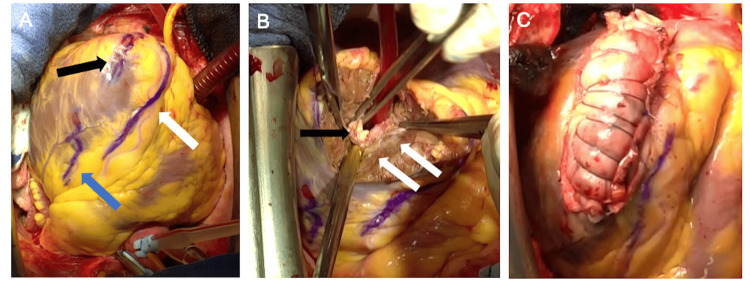
Intraoperative findings (A) A 4 cm incision (black arrow) was made between the left anterior descending branch (white arrow) and the diagonal branch (blue arrow). (B) The apex of the myocardium was scarred (white arrow). A yellow-white thrombus was observed in the trabeculae carneae (black arrow). (C) The left ventricular incision line was reinforced with surgical felt and autologous pericardium.

There was no significant FDG accumulation in the left ventricular apical mass or other organs in FDG-PET CT. The mass in the left ventricle was suspected to be a thrombus based on the findings from imaging studies. Initially, anticoagulation therapy was started with intravenous heparin and oral warfarin. Despite four weeks of warfarin treatment, no reduction in the size of the thrombus was observed. Considering the hypereosinophilia, we performed right ventricular myocardial histopathology. DFS staining revealed eosinophils in the endocardium. Tenascin C, which appears during tissue repair after inflammation, was observed in the endocardium and the interstitium just below the endocardium. These results confirmed the diagnosis of Loeffler’s endocarditis. Because of the mobility of the thrombus and the potential risk of embolism, we opted for thrombectomy without preceding steroid therapy. The approach involved median sternotomy, cardiopulmonary bypass with the ascending aorta, and bicaval venous cannulation. Because the thrombus was entangled in the papillary muscle and trabeculae carneae, it was difficult to remove the thrombus via a transmitral approach, and we chose a trans-left ventricular approach. A 4 cm incision was made between the left anterior descending branch and the first diagonal branch at the apex of the heart. A mixture of normal and scarred myocardial tissue was observed under the endocardium. A thrombus was found in the left ventricular cavity, which was removed as completely as possible while taking care not to damage the papillary muscle. The scarred myocardial tissue was also removed. Intraoperative rapid histopathology revealed no tumor cells, fibrotic left ventricular myocardium, or thrombus. The left ventricular incision line was reinforced with surgical felt at both ends and closed with mattress sutures of 2-0 polypropylene thread, and a strip of autologous pericardium was applied over the incision line with Hydrofit® (Sanyo Chemical Industries, Kyoto, Japan) and reinforced with overhanging sutures (Figure 4). Detachment via cardiopulmonary bypass was easily achieved.

Intraoperative specimen histopathology revealed eosinophilic infiltration of the myocardial tissue. Fibrosis was observed in the perivascular and interstitial areas of the myocardium, and tenascin C was detected, indicating a tissue repair process. These results were consistent with those of Loeffler’s endocarditis. Echocardiography revealed localized hypokinesis in the left ventricular apex, similar to that observed on preoperative echocardiography, but overall cardiac function was well preserved. On postoperative day 1, anticoagulation therapy with heparin and warfarin was resumed, and warfarin was continued with a target PT-INR of 2.0. The patient was discharged on postoperative day 10 with a favorable recovery. While no eosinophilia was evident immediately after surgery, eosinophilia was observed again one month after surgery. The patient was readmitted to our hospital, and a 30 mg dose of prednisone (0.5 mg/kg/day) was started. Following steroid introduction, a prompt decrease in the eosinophil count was observed. Prednisone was gradually tapered to 15 mg, after which the patient was discharged. The prednisone dose was reduced to 5 mg, and the treatment is currently being continued. Six months after surgery, the eosinophil count remained within the normal range (Figure 1), and there was no deterioration in cardiac function or recurrence of left ventricular thrombus.

## Discussion

Since Loeffler and colleagues reported myocardial fibrosis from eosinophilic infiltration into the myocardium in 1936, numerous cases of Loeffler’s endocarditis or eosinophilic endomyocarditis have been reported [1-4]. Although hypereosinophilic syndrome (HES) is considered to cause organ damage, it is an extremely rare disease with an incidence of 0.036 per 100,000 individuals [5]. Approximately 60% of patients with HES experience Loeffler’s endocarditis [6]. In HES, eosinophils increase primarily or secondarily, and the diagnostic criterion includes an eosinophil count >1,500/μL in two or more blood samples taken at intervals of more than a month [3]. Eosinophils infiltrate myocardial tissue, and the release of basic proteins and reactive oxygen species from activated eosinophils leads to thrombus formation and fibrosis, eventually causing restrictive cardiomyopathy [5]. Loeffler’s endocarditis, associated with severe heart failure and embolism, is considered a serious condition with a 10-year survival rate of less than 50% [1], emphasizing the importance of rapid diagnosis and treatment.

For diagnosis, multiple modalities are required to exclude cardiac tumors, infective endocarditis, and intracardiac thrombus formation from low cardiac function. In particular, cardiac MRI is useful for diagnosis [3,7-10]. Findings such as the absence of contrast effects in the mass on contrast-enhanced CT or lack of FDG accumulation on PET-CT also contribute to the diagnostic process [9]. In this case, in addition to the imaging findings, several suggestive features of Loeffler’s endocarditis, such as nonspecific negative T waves and asynergy limited to the surrounding thrombus, were observed [6]. In patients suspected of having Loeffler’s endocarditis, myocardial biopsy is essential for establishing a definitive diagnosis.

Antithrombotic treatment with warfarin and anti-inflammatory treatment with steroids are the standard treatments for Loeffler’s disease-related endocarditis. However, as reported by Ishii and colleagues, thrombus reduction may take a long time, even up to 10 months after initiating warfarin and steroids [2]. An embolism occurs in approximately 30% of patients with Loeffler’s endocarditis who have left ventricular thrombus [3]. Severe thromboembolic events, such as saddle embolism and multiple cerebral infarctions, are frequently reported [1-3,5]; however, there are fewer reports of surgical thrombectomy [4,11]. The main causes of death in this disease are heart failure and embolism [3]; therefore, preventing embolism is a crucial factor in treatment. In addition, surgery with full heparinization after an embolism may lead to hemorrhagic infarction, so surgery should be performed before the embolism occurs. We believe that early thrombectomy should be considered for patients with a mobile thrombus and a risk of embolism.

For preservation of postoperative left ventricular function, a transmitral approach through a left atrial incision is preferable. However, because the thrombus was entangled with the papillary muscles and trabeculae in this patient, a left ventricular approach was chosen. The incision in the region with asynergy did not create new areas of wall motion abnormalities postoperatively. There was no observed decline in cardiac function or occurrence of ventricular arrhythmias following left ventricular incision, suggesting that this approach was safe.

Ikäheimo et al. reported cases where fibrosis of the myocardium progressed after surgery in Loeffler’s endocarditis [11]. In this patient, the eosinophil count, which was initially low after surgery, subsequently increased one month after surgery. This finding suggested that while thrombectomy is useful for preventing thromboembolism, it may not be curative for eosinophilic inflammation. Initiating steroid therapy is considered crucial for inhibiting inflammation via eosinophils and preventing the progression of myocardial damage. In previous reports, the common starting dose for prednisone was 1 mg/kg/day [2]. However, in this patient, considering the relatively mild nature of eosinophilic inflammation and considering the risk of infection, we initiated treatment with 0.5 mg/kg/day. After the introduction of prednisone, a rapid decrease in the eosinophil count was observed. Six months after surgery, his eosinophil count remained within the normal range (Figure 1), and no worsening of cardiac function or recurrence of left ventricular thrombus was observed.

Thrombectomy for Loeffler’s endocarditis has rarely been reported, and the present case has only short-term results. However, further investigations and evaluations of long-term outcomes through extended follow-up are needed to assess the utility of thrombectomy.

## Conclusions

In this report, we present a case of surgery for Loeffler’s endocarditis. Thrombosis caused by Loeffler’s endocarditis can lead to fatal embolism, and since drug treatment requires a long period, thrombectomy should be considered for cases with a high risk of embolism. Combining surgery with drug treatments such as steroids allows for more reliable treatment.
